# Supplementary material for: Human and macaque pairs employ different coordination strategies in a transparent decision game
Source: eLife. 2023 Jan 12;12:e81641. doi: 10.7554/eLife.81641 (PMC9937648; doi:10.7554/eLife.81641)
Supplement: Supplementary file 1. [file elife-81641-supp1.pdf]

Pair ID	N solo trials	Solo trials: fraction choosing own, agent A [%]			Solo trials: fraction choosing own, agent B [%]			Choice preference (own/other's/ balanced), last 200 dyadic trials		AT_A, all trials	AT_B, all trials	Test on AT_A vs. AT_B			$AT_A - AT_B$ all trials	$\lvert AT_A - AT_B \rvert$ all trials	$\lvert AT_A - AT_B \rvert$, coordinated trials			$\lvert AT_A - AT_B \rvert$, uncoordinated trials			Test on coordinated vs. uncoordinated AT differences			Total reward [EUR]	
		all trials	26-50	last 25	all trials	26-50	last 25	agent A	agent B	mean	mean	df (corr.)	t	p	mean	mean	mean	SD	N trials	mean	SD	N trials	df (corr.)	t	p	agent A	agent B
column	*2*	*3*	*4*	*5*	*6*	*7*	*8*	*9*	*10*	*11*	*12*	*13*	*14*	*15*	*16*	*17*	*18*	*19*	*20*	*21*	*22*	*23*	*24*	*25*	*26*	*27*	*28*
1	100	43	44	48	54	52	60	bal.	bal.	493.2	598.7	793.2	-21.1	**<0.0001**	-105.5	113.7	119.8	77.9	306	94.2	70.5	94	168.6	3.0	**0.0031**	20.0	19.2
2	100	93	**100**	**100**	96	**100**	**100**	bal.	bal.	572.1	500.1	597.6	16.5	**<0.0001**	72.0	82.6	83.3	74.3	383	66.8	61.2	17	18.2	1.1	0.2956	22.8	24.0
3	100	86	**88**	**100**	55	60	52	bal.	bal.	672.6	560.0	669.1	17.2	**<0.0001**	112.6	138.6	151.1	87.6	263	102.1	79.0	90	169.4	4.9	**<0.0001**	19.5	19.0
4	100	92	**100**	**100**	99	**100**	**100**	own	own	519.1	540.3	795.7	-5.9	**<0.0001**	-21.3	42.8	n/a	n/a	0	42.8	36.7	400	n/a	n/a	n/a	14.9	15.0
5	100	98	**100**	**100**	95	**100**	**100**	own	bal.	510.6	534.0	786.6	-7.6	**<0.0001**	-23.4	47.6	45.3	35.6	121	48.6	36.0	279	230.6	-0.8	0.3990	18.6	16.7
6	100	92	**100**	**100**	87	**92**	**92**	bal.	bal.	629.4	635.6	797.8	-1.3	0.1813	-6.2	60.5	60.0	50.0	396	104.3	70.0	4	3.0	-1.3	0.2954	23.8	23.7
7	100	26	32	4	50	64	48	bal.	bal.	469.8	497.4	797.0	-5.1	**<0.0001**	-27.6	63.4	61.4	47.0	367	85.5	53.3	33	36.6	-2.5	**0.0165**	21.6	22.4
8	100	54	56	52	99	**100**	**100**	bal.	bal.	454.6	625.2	795.9	-31.6	**<0.0001**	-170.6	174.4	179.3	76.5	377	94.5	84.4	23	24.3	4.7	**0.0001**	23.1	23.0
9	100	97	**100**	**100**	93	**100**	**100**	bal.	bal.	541.1	537.4	663.6	0.8	0.4098	3.7	56.8	56.5	61.0	320	57.9	51.1	80	140.9	-0.2	0.8250	22.0	22.0
10	50	86	**96**	**96**	24	16	16	own	other's	512.8	548.6	554.1	-8.1	**<0.0001**	-35.8	62.0	61.7	54.3	300	156.2	0.0	1	n/a	-30.2	n/a	19.4	14.7
11	50	46	48	48	2	0	0	bal.	other's	478.7	517.8	580.6	-10.5	**<0.0001**	-39.1	56.8	59.9	51.7	176	52.5	37.6	124	297.7	1.4	0.1521	13.6	10.5
12	50	34	64	64	61	64	64	bal.	bal.	577.3	599.9	597.9	-2.9	**0.0039**	-22.6	90.2	91.5	89.2	276	75.3	63.6	24	31.5	1.2	0.2586	15.7	17.2
13	50	89	**92**	**92**	60	44	44	bal.	bal.	616.1	516.3	549.8	16.0	**<0.0001**	99.8	117.3	123.6	89.0	239	92.7	64.5	61	125.1	3.1	**0.0026**	15.8	15.1
14	50	94	**100**	**100**	50	40	40	own	other's	470.9	505.9	596.4	-10.2	**<0.0001**	-35.1	49.7	50.9	43.9	240	45.0	43.6	60	91.2	0.9	0.3562	18.7	13.7
15	100	1	0	0	53	44	40	other's	bal.	468.6	436.7	769.2	8.1	**<0.0001**	31.9	58.9	71.8	53.4	166	49.8	47.3	235	327.6	4.2	**<0.0001**	13.1	16.3
16	100	58	44	**80**	20	40	0	own	other's	543.3	544.0	795.7	-0.2	0.8444	-0.8	50.2	48.2	45.7	388	115.4	83.2	12	11.2	-2.8	**0.0174**	26.0	19.6
17	100	94	**100**	**100**	94	**96**	**100**	bal.	bal.	785.0	664.4	786.3	21.6	**<0.0001**	120.6	129.8	123.3	72.1	337	164.9	62.5	63	95.6	-4.7	**<0.0001**	22.4	22.5
18	100	96	**96**	**96**	31	48	24	other's	own	607.4	702.9	537.3	-15.5	**<0.0001**	-95.4	114.9	115.3	88.1	398	46.3	13.5	2	1.5	6.6	**0.0455**	21.0	25.9
19	100	93	**100**	**100**	1	4	0	own	own	614.9	592.7	787.7	4.2	**<0.0001**	22.2	68.4	n/a	n/a	0	68.4	63.7	407	n/a	n/a	n/a	15.1	13.7

Supplementary file 1. Table S1: human pairs.

Columns 2-10: No strong relationship between own target preference in solo trials and dyadic behavior. Number of solo trials and percentage of choosing own preferred target color in each agent of each human pair, for all solo trials, trials 26-50, and the last 25 solo trials. In 19 of 38 subjects there was clear preference for their own color at the end of the solo training trials (own color selection >75% in 26-50 and last 25 trials, marked by bold font). Only 4 subjects selected own color in <25% of trials 26-50 and the last 25 solo trials (marked by red font). Note that out of 28 subjects trained with 100 solo trials only one (16A) did not reach 75% threshold in trials 26-50 trials but eventually reached it in trials 76-100 – this shows that 50 solo training trials used in pairs 10-14 was generally sufficient for training. Pairs displaying dynamic coordination (turn-taking behavior) are designated by a teal background. Choice preference in dyadic trials: other's (FCO<25%), balanced ("bal.", 25%≤FCO≤75%), own (FCO>75%). Out of 10 pairs (20 subjects) that showed turn-taking, 11 subjects showed a strong preference for own targets already in solo trials (bold font), and 9 did not. In the remaining 9 pairs (18 subjects), 7 subjects who had own preference in solo trials also showed it in dyadic trials, 4 subjects who had other's preference in solo trials also showed it in dyadic trials, and 5 out of remaining 7 subjects switched the preference from solo to dyadic trials, e.g. other's to own. Thus, the behavior in the solo trials is not predicting the behavior in the dyadic trials. This indicates that most human participants did not simply carry their established preferences into the dyadic setting, but continued to explore and modify their strategies. **Columns 11-26: Action times, action time differences and coordination.** Columns 13-15: comparison of ATs between the two players by t-test using Satterthwaite's approximation to allow for unequal variance. Columns 18-26: comparison of absolute trial-by-trial AT differences between the two agents in the pair ($\lvert AT_A - AT_B \rvert$, note that this is equivalent with the difference in AT between the slower and the faster agent individually for each trial). The t-test using Satterthwaite's approximation showed significant differences between coordinated and uncoordinated trials in 9 pairs (bold green and purple font denotes significant differences with a p<0.05; green shows pairs in which absolute AT differences increased with coordination - indicating that seeing the other's action can facilitate coordination, purple – pairs in which absolute AT differences decreased with coordination). Pairs 4, 10, and 19 had too few trials for one of the trial types to calculate the test statistic (n/a). **Columns 27-28. The total reward earned by each agent in solo and dyadic trials**. Note that pairs 10-14 were offered only 350 (50 solo and 300 dyadic) trials instead of 500 (100 solo and 400 dyadic) trials, explaining the lower totals.
