# Supplementary material for: Human and macaque pairs employ different coordination strategies in a transparent decision game
Source: eLife. 2023 Jan 12;12:e81641. doi: 10.7554/eLife.81641 (PMC9937648; doi:10.7554/eLife.81641)
Supplement: Supplementary file 2. [file elife-81641-supp2.pdf]

Pair	Strategy	Macaque A or Conf.	Conf. trained	Macaque B	Conf. trained	Start date	End date	AT_A, all trials	AT_B, all trials	Test on AT_A vs. AT_B			$AT_A – AT_B$ all trials
								mean	mean	df (corr.)	t	p	mean
column	*2*	*3*	*4*	*5*	*6*	*7*	*8*	*9*	*10*	*11*	*12*	*13*	*14*
FC	F's color	F		C		20171019	20171103	489.6	517.4	580.2	-4.7	**<0.0001**	-27.8
MC	M’s left side	M		C		20171108	20180214	509.4	609.1	797.3	-13.0	**<0.0001**	-99.7
MF	M’s left side	M		F		20171129	20180125	444.2	444.9	972.1	-0.2	0.8242	-0.6
Conf-C	Blockwise turn-taking	Conf		C		20171206	20180423	589.6	655.8	303.6	-6.4	**<0.0001**	-66.2
Conf-F	Blockwise turn-taking	Conf		F		20180131	20180228	628.9	752.4	704.8	-13.7	**<0.0001**	-123.5
FC-ct	Temporal competition	F	yes	C	yes	20180418	20180427	573.1	592.5	1091.8	-3.3	**0.0011**	-19.3
TF	T's color	T		F	yes	20180504	20180509	493.0	769.9	415.9	-37.9	**<0.0001**	-276.9
TE	T’s left side	T		E		20180516	20180524	451.6	588.8	509.5	-24.4	**<0.0001**	-137.2
TC	T's color	T		C	yes	20180525	20180530	522.2	715.5	416.5	-21.0	**<0.0001**	-193.3
CE	C’s left side	C	yes	E		20180605	20180615	544.8	694.5	1001.5	-26.5	**<0.0001**	-149.7
LE	L's color	L		E		20181023	20181120	491.7	531.7	860.0	-10.7	**<0.0001**	-40.0
CL	L's color	C	yes	L		20181127	20181211	609.1	460.1	853.2	42.4	**<0.0001**	149.0

Supplementary file 2. Table S2: sequence of macaque pairings and confederate training sorted by the start date, and action times.

Columns 2-8: For each pair we show the joint strategy they converged on and the confederate-training status of each macaque agent (FC-ct: pair FC, confederate-trained, teal background). Macaque pairs converged on static coordination strategies even after one monkey in a pair has been confederate-trained. The confederate training however might have increased the willingness of the trained monkey to accommodate (in all 3 cases of static coordination on fixed color, the confederate-trained monkey followed to the partner’s color: pairs TF, TC, CL). **Columns 9-14: Action times, action time differences, and the t-test on the mean AT differences between the two agents in each pair.** All 5 pairs coordinating on the static color converged on the faster monkey’s preferred color. Likewise, for the static side coordination, all 3 (out of 4) pairs where there was a significant difference in action times between the two agents converged on the faster monkey’s less effortful side closer to the acting arm. This shows that the static coordination in macaques was shaped by the faster agent’s preferences.
