# Supplementary material for: Human and macaque pairs employ different coordination strategies in a transparent decision game
Source: eLife. 2023 Jan 12;12:e81641. doi: 10.7554/eLife.81641 (PMC9937648; doi:10.7554/eLife.81641)
Supplement: Supplementary file 3. [file elife-81641-supp3.pdf]

Session	df	r(A)	r(B)	p(A)	p(B)	raw r(A)	raw r(B)	raw p(A)	raw p(B)
Prior to confederate training (naïve pair)									
FC-1	304	-0.544	0.169	**5.12E-25**	**0.002952**	-0.068	0.043	0.23521	0.45327
FC-2	365	0.236	-0.536	**4.62E-06**	**1.17E-28**	0.388	-0.114	**1.27E-14**	**0.028859**
FC-3	183	-0.351	0.200	**9.42E-07**	**0.006239**	-0.124	-0.062	0.092404	0.40478
FC-4	207	-0.376	-0.200	**2.00E-08**	**0.003778**	-0.128	0.218	0.064765	**0.001527**
FC-5	171	0.194	0.161	**0.010527**	**0.034044**	0.111	-0.014	0.14464	0.85860
FC-6	397	n/a	-0.096	n/a	0.054399	n/a	-0.039	n/a	0.44066
FC-7	314	-0.044	0.560	0.433	**1.68E-27**	0.024	0.144	0.67187	**0.010417**
FC-8	447	0.447	0.228	**1.67E-23**	**9.91E-07**	0.171	0.027	**0.00027874**	0.5692
During confederate training									
F-1	431		0.063		0.19125		0.079		0.10121
F-2	714		0.125		**0.000804**		0.108		**0.003798**
F-3	578		-0.053		0.20033		0.140		**0.000699**
F-4	80		0.040		0.72184		0.248		**0.024749**
F-5	547		0.044		0.30683		0.173		**4.56E-05**
F-6	317		0.280		**3.54E-07**		0.205		**0.000225**
F-7	540		0.141		**0.001023**		0.170		**6.82E-05**
F-8	582		0.077		0.063123		0.076		0.068242
F-9	547		0.260		**5.64E-10**		0.298		**9.42E-13**
F-10	479		0.544		**2.19E-38**		0.390		**6.99E-19**
F-11	473		0.820		**7.89E-117**		0.586		**3.44E-45**
F-12	525		0.829		**1.15E-134**		0.579		**1.40E-48**
F-13	594		0.815		**3.75E-143**		0.589		**7.59E-57**
F-14	554		0.867		**2.42E-169**		0.557		**1.10E-46**
F-15	446		0.868		**1.40E-137**		0.642		**2.38E-53**
C-1	652		0.161		**3.51E-05**		0.188		**1.37E-06**
C-2	544		0.311		**9.50E-14**		0.172		**5.18E-05**
C-3	320		0.482		**3.56E-20**		0.307		**1.78E-08**
C-4	455		0.145		**0.001896**		0.188		**5.03E-05**
C-5	412		0.588		**7.67E-40**		0.285		**3.63E-09**
C-6	476		0.697		**7.88E-71**		0.408		**1.46E-20**
C-7	581		0.670		**3.11E-77**		0.388		**2.42E-22**
C-8	228		0.715		**2.36E-37**		0.471		**4.42E-14**
After confederate training (trained pair)									
FC-1	371	0.806	0.834	**2.45E-86**	**1.24E-97**	0.516	0.603	**8.13E-27**	**3.13E-38**
FC-2	545	0.916	0.909	**3.57E-218**	**1.28E-209**	0.635	0.655	**4.15E-63**	**2.93E-68**
FC-3	625	0.753	0.518	**6.88E-116**	**2.65E-44**	0.423	0.321	**1.19E-28**	**1.76E-16**
FC-4	471	0.856	0.517	**9.73E-137**	**9.49E-34**	0.420	0.305	**1.20E-21**	**1.22E-11**
FC-5	683	0.707	0.654	**1.16E-104**	**9.32E-85**	0.562	0.430	**2.71E-58**	**3.53E-32**
FC-6	370	0.901	0.822	**3.33E-136**	**1.29E-92**	0.601	0.580	**7.55E-38**	**7.34E-35**

Supplementary file 3. Table S3: action time correlations in macaque pair FC.

Correlation between the probability to see other's action and the selection of the other's target. Prior to confederate training (8 naïve sessions), monkeys F (agent A) and C (agent B) showed only relatively weak and inconsistent (both positive and negative) correlations between probability to see other's action (r(A) and r(B)) and the selection of the other's target. Correlation degree of freedom (df), correlation coefficients (r) and correlation p-values (p) for the running average over 8 trials as displayed in **Figure 6 – figure supplement 1**, and for the raw non-smoothed data are shown. Bold font denotes correlations with $p<0.05$. n/a – not applicable: the correlation is undefined because at least one variable was constant (e.g. the fraction of choosing other's 0). The few high correlations on smoothed data were mainly driven by the slow changes in the timecourse of the session. During confederate training both monkeys (F, C) developed strong and highly significant positive correlations between being able to see the confederate's actions and following the confederate: 15 sessions of monkey F and 8 sessions of monkey C during confederate training. Note the human confederate worked on position A, and is excluded here. Bold font denotes correlations with a $p<0.05$. After confederate training this pair continued to show strong correlations between seeing the other's choice and following: 6 sessions of monkeys F and C after confederate training. Note that even in the sessions without significant DCR in the last 200 trials (i.e. FC-1, FC-3, FC-4, FC-6), where monkeys largely converged on one color, the high positive correlations reflect brief bouts of competitive turn-taking or challenging.
