# Supplementary material for: Human and macaque pairs employ different coordination strategies in a transparent decision game
Source: eLife. 2023 Jan 12;12:e81641. doi: 10.7554/eLife.81641 (PMC9937648; doi:10.7554/eLife.81641)
Supplement: Supplementary file 4. [file elife-81641-supp4.pdf]

Pair ID	df	r(A)	r(B)	p(A)	p(B)	raw r(A)	raw r(B)	raw p(A)	raw p(B)
4	398	n/a	n/a	n/a	n/a	n/a	n/a	n/a	n/a
19	405	n/a	n/a	n/a	n/a	n/a	n/a	n/a	n/a
15	399	-0.123	-0.110	**0.014003**	**0.026945**	0.067	0.176	0.1802	**0.000399**
11	298	-0.097	n/a	0.09230	n/a	0.040	n/a	0.48759	n/a
5	398	-0.009	-0.427	0.85640	**3.93E-19**	-0.028	-0.171	0.58233	**0.000598**
3	351	0.164	0.006	**0.001941**	0.91763	-0.012	0.054	0.82342	0.30902
1	398	-0.009	0.025	0.85616	0.62476	0.027	0.026	0.59697	0.60243
14	298	0.060	0.079	0.29914	0.17498	0.010	0.154	0.86265	**0.007371**
13	298	0.126	0.108	**0.029495**	**0.061976**	0.026	-0.037	0.65319	0.52015
2	398	0.243	0.394	**9.12E-07**	**2.54E-16**	0.095	0.124	0.05769	**0.012791**
12	298	0.523	0.456	**1.98E-22**	**8.08E-17**	0.268	0.330	**2.41E-06**	**4.90E-09**
16	398	0.046	-0.569	0.36308	**1.18E-35**	0.142	-0.214	**0.004470**	**1.64E-05**
7	398	0.053	0.287	0.29108	**4.94E-09**	0.001	0.066	0.97791	0.18541
9	398	0.126	-0.271	**0.011469**	**3.59E-08**	0.009	-0.025	0.85404	0.61258
17	398	-0.487	0.187	**3.10E-25**	**0.000171**	-0.054	0.038	0.28453	0.44647
8	398	-0.114	0.075	**0.022805**	0.13386	-0.036	0.055	0.47255	0.2706
6	398	-0.370	0.071	**2.04E-14**	0.15713	0.040	0.017	0.41953	0.73857
10	299	0.251	n/a	**1.07E-05**	n/a	0.221	n/a	**0.000109**	n/a
18	398	0.048	0.176	0.34150	**0.000399**	-0.000	0.094	0.99829	0.06041

Supplementary file 4. Table S4: action time correlations in humans.

Correlation between the probability to see other's action and the selection of the other's target in 19 human pairs. Human pairs showed weak and inconsistent (both positive and negative) correlations between probability of seeing the other's choice and selecting the other's target. Correlation degree of freedom, correlation coefficients and correlation p-values for the running average over 8 trials, as displayed in **Figure 6 – figure supplement 1**, and for the raw non-smoothed data are shown. Letters (A) and (B) denote the positions of the agents. Pairs are sorted by the average joint reward in ascending order (cf. **Figure 2 – figure supplement 2B**). Pairs displaying dynamic turn-taking behavior are designated by a teal background. Bold font denotes correlations with a $p<0.05$. n/a – not applicable: the correlation is undefined because at least one variable was constant (e.g. the fraction of choosing other's 0).
