# Supplementary material for: Human and macaque pairs employ different coordination strategies in a transparent decision game
Source: eLife. 2023 Jan 12;12:e81641. doi: 10.7554/eLife.81641 (PMC9937648; doi:10.7554/eLife.81641)
Supplement: Supplementary file 5. [file elife-81641-supp5.pdf]

Appendix 1

Supplementary Materials and Methods

Written instructions read by human subjects

Instructions read to human subjects by experimenter

Debriefing questions

Abbreviations

Written instructions read by human subjects

Description of the study

This study is the investigation of the behavioral correlates of social decision making while playing a game with a partner. Every day we have to make decisions that depend not only on our own needs and goals but also on the needs and goals of others: for instance, while working on a project with colleagues, planning vacation with friends and family, getting on the bus or shopping groceries, etc. With the help of the task presented in this study we will investigate how people make such decisions.

The course of the study

You will complete one session of a decision making task on the computer together with your partner. You will have to choose one of the two circles presented to you. Your partner will have to perform the same task on his/her side of the touchscreen. You will have to decide and respond quickly. If either you or your partner are too slow, the trial will be aborted without any reward. Your reward will depend on your own and your partner’s choice. After the decision is made, you both will receive different auditory feedbacks, denoting your reward and the reward of your partner. Please do not talk to your partner during the session. After the session, we will ask you several questions about the experiment.

Instructions read to human subjects by experimenter

1) You rest both hands on the gray board at the two round objects (touch sensors).
2) A central touch target will appear.
3) Move your right hand to the target and hold. Use the right hand during the entire session.
4) While the target brightens up keep holding your finger on the target.
5) Then two colored choice targets appear and the central target disappears.
6) Make your choice and touch the chosen target within 1.5 seconds.
7) All touched targets will brighten up, keep holding until the targets disappear.
8) Please note that both selected targets will brighten up:
   In case both players selected the same target only that single target will brighten;
   In case both players selected different targets, both targets will brighten.
9) Now two streams of auditory beeps will signify the earned reward for each player: each beep corresponds to a few cents. Please try to learn the sound related to your reward during the training trials.
10) While the audio plays move the hand back to the two touch sensors.
11) Go to 1.

Debriefing questions

1) Was there any recognizable system between the choice item and reward size?
2) How did you make your choice which circle to choose?
3) How do you think the other player decides which circle to choose?
4) Do you think there is an optimal strategy, if so, what?
5) Did the possibility to sit in front of the partner have any impact on your choices?
6) Rate the cooperativity of your partner, with 1 fully cooperative and 7 fully competitive.
7) Rate the cooperativity of yourself, with 1 fully cooperative and 7 fully competitive.
8) How did you recognize the other player's choice?
9) Did you observe any relation between colors and reward magnitude per trial?

Abbreviations

AT	Action time
BoS	Bach-or-Stravinsky game (also known as the Battle of the Sexes)
DCR	Dynamic coordination reward
FCL	Fraction of choosing left
FCO	Fraction of choosing own
MI	Mutual information
MIS	Mutual information between side choices
MIT	Mutual information between target color choices
(i)PD	(iterated) Prisoner’s dilemma
SD	Standard deviation
